# ApoE COG 133 mimetic peptide improves survival, infection burden, and *Clostridioides difficile* toxin-A-induced intestinal damage in mice

**DOI:** 10.3389/ebm.2025.10638

**Published:** 2025-12-03

**Authors:** Orleâncio G. R. de Azevedo, Jae H. Shin, Rosemayre S. Freire, Gabriella C. V. Ciurleo, Gerly A. C. Brito, Michael P. Vitek, Richard L. Guerrant, Reinaldo B. Oriá, Cirle A. Warren

**Affiliations:** 1 Laboratory of the Biology of Tissue Healing, Ontogeny and Nutrition, Department of Morphology and Institute of Biomedicine, School of Medicine, Federal University of Ceara, Fortaleza, Brazil; 2 Division of Infectious Diseases and International Health, School of Medicine, University of Virginia, Charlottesville, VA, United States; 3 Analytical Core Facility, Federal University of Ceara, Fortaleza, Brazil; 4 Department of Morphology, Faculty of Medicine, Federal University of Ceara, Fortaleza, Brazil; 5 Department of Neurology, Duke University Medical Centre, Durham, NC, United States

**Keywords:** *C. difficile* infection, toxin a, apolipoprotein E mimetic peptide, ApoE knockout, intestinal inflammation

## Abstract

Apolipoprotein E (ApoE = protein; APOE = gene), a lipid carrier that modulates inflammatory responses, may influence *Clostridioides difficile* (*C. difficile*) infection (CDI) outcomes. We explored the role of the APOE gene using apoE-deficient mice challenged by *C. difficile* toxin A (TcdA)-induced enteritis, and the potential use of the ApoE mimetic peptide in repairing the intestinal damage induced by TcdA. 4-cm ileal loops from C57BL/6 wild-type and APOE knockout (−/−) were ligated and injected with either PBS or TcdA (50 µg). After 4 h of incubation, the intestinal loops were harvested for measurement of length, weight, volume of secretion, and histopathology scores. In mouse ileal loops, TcdA induced a significant increase in weight/ileal loop length in the wild-type mice. When APOE^−/−^ mice were infected with 1 × 10^4^–10^5^ CFUs of *C. difficile*, they had higher deaths and diarrhea scores compared to wild-type. APOE^−/−^ mice under the toxin A (TcdA) had worse inflammatory changes in the ileal loop. APOE^−/−^ mice treated with COG133 (3 mg/kg) showed fewer deaths, and lower diarrhea scores, but no change in *C. difficile* shedding. This suggests a potential anti-inflammatory role of COG133 in CDI. More studies are neede to these intial findings in depth.

## Impact statement

This short communication brings new data that fills a gap in the scientific literature, showing that apoE deficiency during *C. difficile* infection worsens intestinal inflammation. We have also shown that this is partially rescued by COG 133 treatment, an ApoE mimetic peptide COG 133. It was able to reduce the inflammation pattern in *C. difficile*-infected mice and improve survival rates. The mechanism of action of these effects is underway in our research group labs.

## Introduction

*Clostridioides difficile* is a Gram-positive anaerobic *bacillus* [1], associated with 15–20% of antibiotic-associated diarrhea in the United States [2]. Clinical manifestations range from asymptomatic to severe pseudomembranous colitis and death. There has been an increase in *C. difficile* infection (CDI)-related hospitalizations in the USA over time [3]. In addition, the death rate was higher in hospitalizations due to CDI compared to other diseases [3]. The excess costs for CDI-related inpatient healthcare are estimated at $ 4.8 billion and are likely will rise in the upcoming years [4, 5].

*C*. *difficile* pathogenic strains produce two high molecular weight exotoxins: toxin A (TcdA) and toxin B (TcdB), which both induce epithelial cell death and loss of cytoskeletal structure in the cell, contributing to colitis by induction of pro-inflammatory cytokines, disruption of cell-to-cell junctions, increased intestinal permeability, and luminal fluid accumulation [6, 7]. TcdA is a 308-kDa cytotoxin and enterotoxin that induces intestinal epithelial injury, leading to the release of inflammatory mediators [7]. In addition, TcdA has pro-inflammatory and pro-secretory activities [4, 5, 8, 9].

Brayan et al., study the effects of TcdA and TcdB in murine model of intoxication where the subjects had their polymorphonuclears depleted by 100ug of Ly6G/Ly6C and then murine-ligated-ileal loop model [10]. The mice with a regular number of PMN was given TcdA and TcdB showed high levels of IL-1β, IL-6 and TNF-α production. However, the study showed a reduction in cytokine production when TcdA + TcdB were administered together. IL-1β and Il-8 are produced in higher levels, as innate immune inflammatory mediators, in patients with CDI [11]. In general, both toxins induce inflammatory cytokines entering the host cells through endocytosis and then translocating the glucosyltransferase domain and glycosylates proteins such as Rho proteins leading to a disruption of cytoskeleton inducing cell death activating immune cells which synthesizes and release pro-inflammatory cytokines [12]. TcdA is cytotoxic to the intestinal epithelial lining, leading to cytoskeletal protein disruption, loss of epithelial tight junctions, and consequently increased intestinal permeability. Intestinal epithelial barrier damage contributes to a proinflammatory state, activating immune cells to release IL-1β, IL-6, TNF-α, and IL-8, with tissue infiltration of neutrophils and mast cells, initiating a mucosal inflammatory response. TcdA induces COX-2 and PGE2-associated secretory response in the gut by increasing CFTR and calcium-activated chloride channel activity [13].

Apolipoprotein E (ApoE) is a lipid carrier shown to modulate inflammatory and immune responses. ApoE delivers cholesterol to the liver to be metabolized, playing a pivotal role in lipid homeostasis [14]. The ApoE COG133 (133–149) mimetic peptide lacks a lipid-binding role, but it has potent anti-inflammatory properties in models of brain injury and retains the ApoE holoprotein’s neuroprotective roles [15, 16]. According to the study of Li et al., the treatment of mice under a murine experimental autoimmune encephalomyelitis with the administration of COG 133 significanlty reduced the activation of macrophages, production of nitric oxide and pro-inflammatory cytokines, and lymphocyte proliferation [14]. Prior studies have shown that ApoE mimetic peptides inhibited the nitric oxide synthase (iNOS), chemokines, and the NF-KB pathway in a colitis murine model of *C. rodentium* [17] and reduced LPS-induced vascular adhesion molecule-1 and monocyte adhesion to endothelial cells [18]. Our group has documented these COG 133 beneficial effects in reducing intestinal inflammation in mice and hastening IEC-6 cell monolayers' restitution following 5-fluorouracil-induced challenge [19].

In this report, we investigate whether ApoE deficiency could worsen CDI outcomes and whether COG 133 could reverse these effects in mice. In this study, we evaluated the effect of TcdA on fluid accumulation and inflammation in the ileal loop mouse model and the effect of infection by *C. difficile* on animal survival, weight loss, and diarrhea in APOE knockout (APOE ^−/−^) and wild-type mice. No studies have yet evaluated the effects of ApoE deficiency and the COG 133 following CDI.

## Materials and methods

### Reagents and materials

The ApoE mimetic peptide (COG133) was provided courtesy of Michael P. Vitek, (Cognosci Inc., Duke University (Durham, NC) [20]. ApoE COG133 mimics residues 133–149 of the receptor-binding component of the apolipoprotein that binds to ApoE receptors. The ApoE COG 133 amino terminus was acetylated, and the carboxyl terminus was blocked with an amide moiety. The ApoE COG 133 was frozen and reconstituted in sterile PBS for administration during experiments.

Intravenous solutions of vancomycin hydrochloride (Hospira, Inc., Lake Forest, IL), colistimethate (Colistin) (X-GEN Pharmaceuticals, Inc., Big Flats, NY), gentamicin sulfate (Hospira), metronidazole (Flagyl) (Baxter Healthcare Corporation, Deerfield, IL), and cleocin phosphate (clindamycin) (Pfizer New York, NY). *C. difficile* strain VPI10463 was purchased from the American Type Culture Collection (Manassas, VA). Purified Toxin A from *Clostridioides difficile* (strain # 10463; molecular weight 308 kDa) was kindly provided by Dr. David Lyerly (Tech Lab, Blacksburg, VA) [21] and used diluted in PBS (pH≅7.4). Chopped Meat Broth (CM, catalog AS-811) was purchased from Anaerobe Systems (Morgan Hill, CA).

### Mouse ileal loops

C57BL/6 wild type (WT) and APOE knockout (APOE ^−/−^) underwent midline laparotomy, and one 4 cm-ileal loop per mouse was ligated and injected with either 0.1 mL PBS or TcdA (50 µg) (TechLab, Blacksburg, VA) [21]. The abdomen was surgically closed for incubation. Ileal loop ligation was subsequently performed on WT mice, which were injected with ApoE peptide (3 mg/kg) either intraperitoneally or intraluminally, in addition to TcdA, to determine which administration route would improve the TcdA-induced ileal secretion. After 4 h of incubation, the mice were euthanized, and the ileal loops were harvested for measurement of length, weight, and histopathology, which was measured on a scale of 0–3 based on the presence of inflammatory cells, vascular congestion, architectural destruction, and edema [22].

### Infection model

#### Preparation of inoculum

*C. difficile* was grown for 20 h in chopped meat broth (37 °C), transferred to a fresh tube of chopped meat broth, and incubated for 5 h to achieve log-phase growth before infection. Bacteria were centrifuged at 10,000 rpm for 2 min, washed three times with BHI, and then reconstituted in BHI medium. Using spectrophotometry, an OD reading of 1.0 was calculated to be equivalent to 5 × 10^7^ CFU *C. difficile*/mL. The final concentration of *C. difficile* (CFU/mL) was validated by hemocytometer reading under light microscopy. Unless the dose was specifically stated, 1 × 10^4−^10^5^ of *C. difficile* was given by gavage to each mouse in the infected groups, and BHI medium only was given to mice in the uninfected groups.

The infection model is a modification of the published protocol by Chen et al [23]. This protocol has been approved by the Center for Comparative Medicine at the University of Virginia. Approximately 8-week-old C57BL/6 and APOE ^−/−^ mice from Jackson Laboratory (Bar Harbor, ME) were used. APOE ^−/−^ mice were generated from the B6.129APOE ^−/−^ mice at the N10 backcross and constructed from B6.129APOE^−/−^ mice. Sterilized food and water were given *ad libitum* to all mice. Between 4 and 6 days before infection, mice were given an antibiotic cocktail containing vancomycin (0.0045 mg/g), colistin (0.0042 mg/g), gentamicin (0.0035 mg/g), and metronidazole (0.0215 mg/g) by gavage. One day before infection, clindamycin (32 mg/kg) was injected subcutaneously. This antibiotic cocktail was used to facilitate *C. difficile* toxin effect in mice that underwent antibiotic pre-treatment designed to deplete the resident gut microbiota. This combination targets both Gram-positive/negative bacteria and effectively reduces microbial diversity in the intestinal tract. On the day preceding *C. difficile* challenge, mice were administered a single subcutaneous injection of clindamycin (32 mg/kg), a well-established antibiotic in murine models of antibiotics against *C. difficile* infection, and a critical step known to suppress residual microbial populations further and enhance susceptibility to *C. difficile* toxins.

Infection was performed with *C. difficile* strain VPI 10463 inoculum given by gavage. In the first experiment, mice were divided into 4 groups consisting of control uninfected WT mice, control uninfected APOE KO mice, infected WT mice, and infected APOE^−/−^ mice. The mice were observed for 5 days post-inoculation and euthanized. In the next experiment, the mice were divided into the following groups: control uninfected, control infected, infected treated with vancomycin (50 mg/kg), infected treated with ApoE mimetic peptide-COG 133 (3 mg/kg by ip, given 2 x/day), and infected treated with vancomycin and ApoE peptide. One day post-infection, treated mice were given either vancomycin (50 mg/kg) daily with or without ApoE peptide or ApoE peptide alone for 5 days. The very sick animals were euthanized daily, and all surviving mice were euthanized at day 14.

The ApoE mimetic peptide dose for the ileal loop and the infection model was based on the findings from Pane et al., who evaluated the antimicrobial effect *in vitro* against *S. aureus* ATCC 6538P and a clinical isolate of *P. aeruginosa* strain KK27 [24], who investigated the antimicrobial activity of different ApoE-mimetic peptides. A significant COG 133 antimicrobial activity was seen at a concentration of 3.12 µM (∼0.065 mg/kg); However, to improve bioavailability to mice, we decided to use a higher dose. Other study found good results with 2 mg/kg of the ApoE COG 1410 [25].

### Animal handling and endpoints

As described in a prior study, experimental animals were weighed and scored. Daily stool specimens were collected at regular intervals [22]. The diarrhea scores were assessed according to the following scale of 0–3: 0—well-formed pellets; 1—stick stools adhering to the microtube wall or color change; 2—pasty stools with or without mucus; and 3—watery stools. This scoring method was described elsewhere [20] with some modifications. Cecal and colonic tissues were harvested from all animals at autopsy. Tissues were fixed overnight with Bouin’s solution and stored in 70% ethanol. Hematoxylin & Eosin (HE) staining of samples was performed by the University of Virginia Research Histology Core. Slides were examined using a Leica DFC425 digital camera equipped microscope with Leica Application Suite Version 3.6.0.488 imaging software (Leica Microsystems Inc., Buffalo Grove, IL 60089). Histopathology was scored based on inflammation, mucosal disruption, mucosal hypertrophy, exudate, and submucosal edema, as we have previously described [26].

### DNA extraction and quantification

All stool samples were weighed for sample normalization. Stool DNA was extracted under the modified protocol provided in the QIAamp DNA Stool Mini Kit. Briefly, frozen stool was added to 400 μL of ASL buffer, homogenized by grinding with a wooden stick, vortexed for 15 s before and after heating in a water bath at 82.5 °C for 5 min, centrifuged at 14,000 rpm for 2 min. The remaining steps followed the manufacturer’s directions. Extracted stool DNA was stored at −20 °C before PCR testing.

*C. difficile* DNA was analyzed from extracted stool DNAs with iQ SYBR Green Supermix in a 96-well plate performed at CFX96™ Real-Time PCR Detection System (Bio-Rad). A PCR master mix was prepared with a 23 μL aliquot containing 1 μL each of TcdA forward and reverse primers, 12.5 μL iQ SYBR Green Supermix, and 8.5 μL of H2O purified by the Milli-Q Integral Water Purification System (Millipore Corporation, Billerica, MA). 2 μL of each sample was then added to each well filled with PCR master mix aliquot in a 96-well plate. The PCR parameters were sequentially set for 3 stages: 1× cycle for 5 min at 94.0 °C, 40 × cycle for 30 s each from 94.0 °C to 55.0 °C–72.0 °C, 64 °C × cycle at 62.0 °C for 15 s, and 1× cycle for hold at 25.0 °C. Melt curve data collection and analysis were enabled. Copy numbers of the unknown sample were extrapolated from the standard curve that was generated with the extracted DNA prepared from a known *C. difficile* inoculum. The sequence of the TcdB forward primer was 5′-GGA​GAG​TCA​TCC​AAC​TTA​TAT​G-3′, and the reverse primer was 5′-CCA​CCA​ATT​TCT​TTT​AAT​GCA​G-3′.

### Statistical analysis

Statistical analyses were conducted using GraphPad Prism Version 5.02 software. When a mouse was either found dead or sacrificed due to severe distress, the last recorded body weight was continuously plotted against the body weights of surviving mice. Differences between groups for the entire experimental period were analyzed by 2-Way ANOVA with Bonferroni *post hoc* testing. Survival curves were analyzed using the Log-rank (Mantel-Cox) or Log-rank test for mortality trend.

## Results

### ApoE deficiency and ApoE COG 133 peptide modulated histopathology in TcdA-challenged ileal loops

**FIGURE 1:**
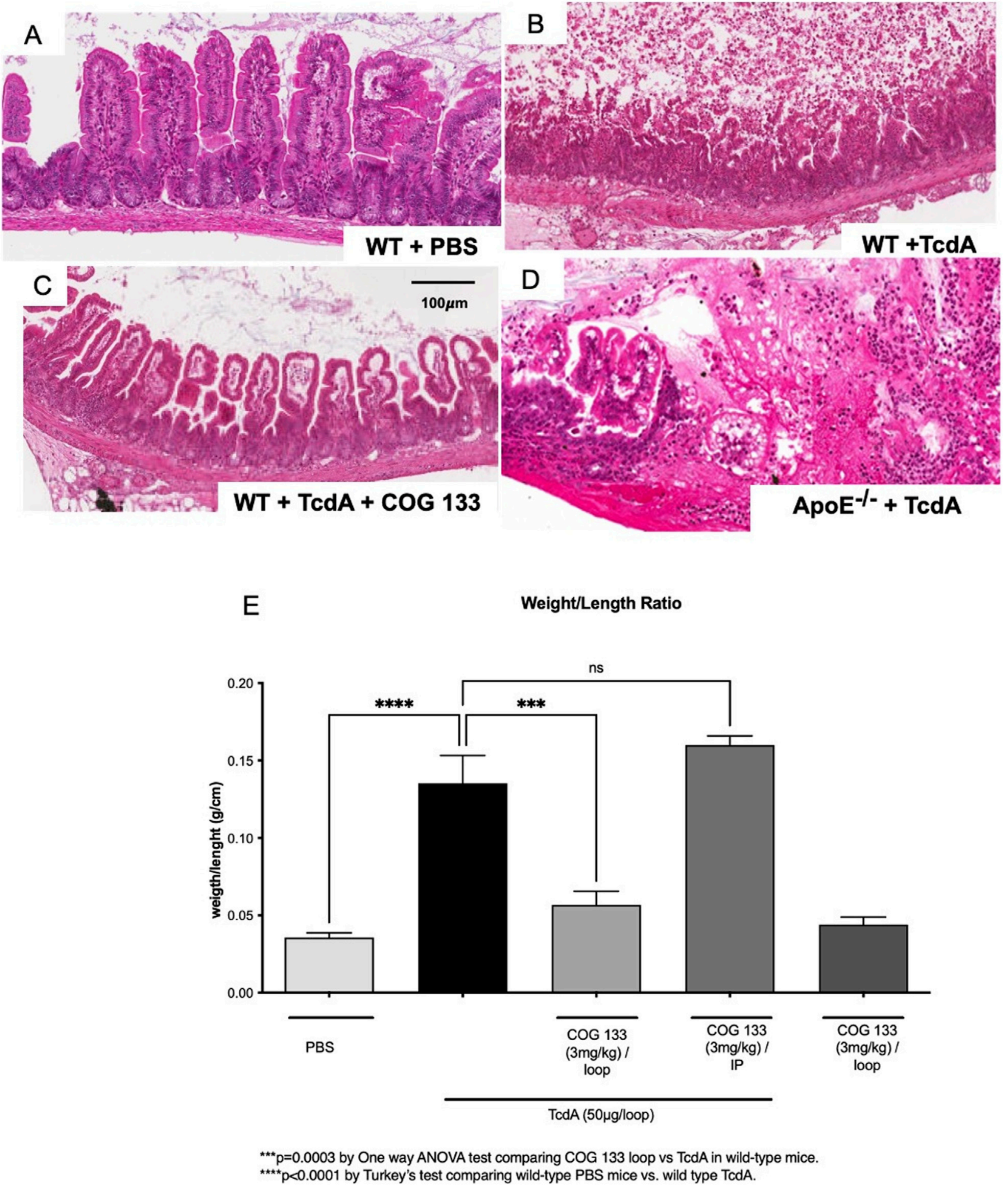
Apolipoprotein E (ApoE) deficiency and COG 133 modulate histopathology in TcdA-challenged ileal loops. Representative histopathology of the intestinal injury of ileal loops from experimental mice. Compared to unchallenged controls **(A)**, *C. difficile*-toxin A (TcdA), 50µg/loop, induced severe intestinal mucosal damage with marked features of inflammation and disrupted villi and crypt glands in wild-type **(B)** and APOE knockout (−/−) mice **(D)**. ApoE COG 133 (COG 133) treatment, at 3 mg/kg, partially rescues intestinal injury following TcdA in wild-type mice **(C)**. Hematoxylin-Eosin staining. Scale bar: 100 µm. TcdA significantly induced an increase in the ileal loop weight/length ratio (g/cm) in wild-type mice compared with intraperitoneal COG 133 treatment **(E)**. Results are expressed by mean ± SD. N = 7 mice per group.

As expected, in wild-type mice, injection of TcdA into the ileal loop caused marked worse intestinal mucosal injury (Figure 1B) and increased weight/length ratio (secretion) (Figure 1E), as compared to wild-type control (Figures 1A,E). These effects were markedly improved by ApoE COG 133 peptide treatment with lower weight/length ratio (p = 0.003) (Figures 1C,E), markedly reducing inflammatory cell infiltrates, and preserving villus and crypt architecture. APOE ^−/−^ mice that received TcdA intraluminally also showed worse histopathology and increased intestinal weight/length ratio (p < 0.0001) (Figure 1D), with marked inflammation, vascular congestion, submucosal edema, and epithelial disruption. However, this beneficial effect was only seen when the peptide was administered intraluminally (Figure 1E).

### ApoE deficiency and ApoE COG 133 peptide influenced the outcome of CDI

**FIGURE 2:**
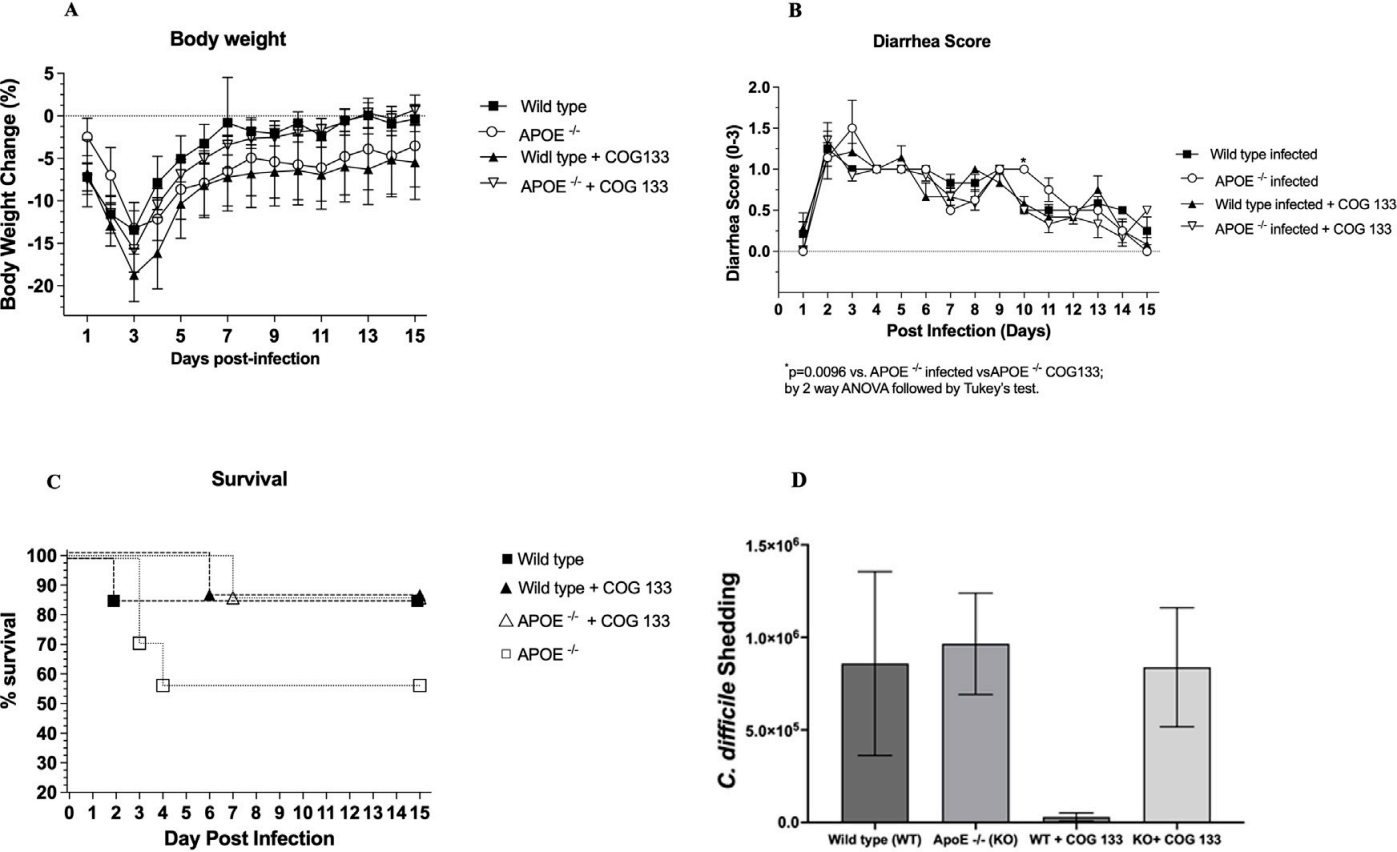
The administration of the ApoE mimetic COG133 peptide (3 mg/kg i.p.) did not affect weight loss **(A)**, however, it significantly reduced diarrhea scores (p = 0.0096) on day 10 post-infection in APOE knockout (−/−) mice subjected to *C*. *difficile* infection (CDI) with the VPI10463 strain (10^5^ CFU by oral gavage) **(B)**. In addition, infected APOE (−/−) mice exhibited absolute less survival rate (57.14%) compared to infected APOE (−/−) treated with COG 133 (85.71%) **(C)**. The *C. difficile* burden measured through qRT-PCR did not document any significant differences between the experimental groups **(D)**. *C. difficile* shedding was expressed in log of C. diff. DNA shedding.

CDI in APOE^−/−^ mice resulted in an absolute number of more deaths than in wild-type infected mice (Figure 2A). Survival in APOE ^−/−^ mice was 57.14% compared to 85.71% in the wild-type infected mice. When treated with COG 133 (3 mg/kg), wild-type and APOE ^−/−^ mice showed survival rates of 87.5% and 85.7%, respectively. These findings suggest that ApoE may confer protection against *C. difficile* infection, leading to a decrease in survival in APOE ^−/−^ mice, which was reversed when ApoE was replaced with COG 133. However, the addition of COG 133 to wild-type mice did not further improve survival. When the weights were compared, APOE ^−/−^ mice did not differ significantly from wild-type mice in weight loss (Figure 2B). When COG 133 was administered in wild-type mice, there were no statistically significant changes in weight changes. When diarrhea scores were compared, APOE ^−/−^ infected mice had significantly higher diarrhea scores, while COG 133 administration significantly improved the diarrhea scores of the APOE ^−/−^ mice on day 10 (p = 0.0096) (Figure 2C).

APOE ^−/−^ mice did not have any significant difference from wild-type mice in *C. difficile* shedding as measured by qPCR. The administration of COG 133 did not show a significant difference in the *C. difficile* shedding in wild-type and APOE^−/−^ mice, suggesting that the peptide COG 133 (3 mg/kg) may have inconsistent activity on *C. difficile* growth (Figure 2D).

## Discussion

This study suggests that ApoE has a modulatory role in the pathogenesis of CDI. The mouse model demonstrated more severe CDI in APOE^−/−^ compared to WT mice, that were partially improved when ApoE mimetic peptide was given. In the mouse ileal loop model utilizing toxin A (TcdA) injection, ApoE deficiency worsened the inflammation patterns on histology and worsened the edema as measured by the weight/length ratio. However, the intraluminal treatment with COG 133 peptide improved weight/length ratios in animals challenged by TcdA, suggesting that the role of ApoE may be cell or tissue-specific and may be affected by the presence or absence of other inflammatory cells.

The critical role of ApoE in affecting susceptibility to infection was demonstrated using APOE^−/−^ mice in a model of *Mycobacterium smegmatis, Klebsiella pneumoniae,* and *Cryptosporidium parvum* infection [27–29]. In a study evaluating the effect of ApoE against gram-negative bacteria, ApoE exhibited anti-bacterial activity against *P. aeruginosa* and *E. coli in vitro* and an anti-inflammatory activity against *P. aeruginosa and E. coli* infection in a mouse model, mediated by binding LPS and reducing the inflammation in those infected mice [29]. ApoE has been shown to directly regulate the type 1 inflammatory response, with significantly greater proinflammatory cytokines such as TNF-α, IL-6, IL-12, and IFN-γ in APOE^−/−^ mice than in WT mice following LPS challenge [30].

The importance of ApoE was also demonstrated by studies utilizing ApoE mimetic peptides [30]. In a *Citrobacter rodentium* infection mouse model, ApoE mimetic peptide COG 112 demonstrated inhibition of NF-κB pathway, blocking chemokines, nitric oxide, and p53 release [17]. The anti-inflammatory activity of COG 112 in *C. rodentium* colitis reduced the inflammatory pattern in ileal samples of APOE ^−/−^ and wild-type infected mice. APOE23, a new combination of two fragments of ApoE 141–148 and ApoE 135–149, demonstrated anti-bacterial activity against *E. coli*, *P. aeruginosa*, *S. aureus*, *Enterococcus faecalis,* and *Acinetobacter baumannii* (MDR). These ApoE peptides also showed anti-inflammatory effects, reducing the levels of expression of TNF-α and IL-6 in THP-1 cells treated with LPS [32].

Another study demonstrated the antimicrobial effect of COG 1410 ApoE mimetic peptide against pan-drug-resistant *A. baumannii.* The mimetic peptide exhibited biofilm inhibition and eradication activity; the peptide induces a lesion in the bacterial membrane. In addition, the COG 1410 controlled the oxidation-reduction processes [33].

ApoE COG 133 has demonstrated protective activity in infection and inflammation models. A study investigating the CM-A (cecropin-A and melittin) that showed antimicrobial activity *in vitro* experiments. The authors demonstrated the biological role of the peptides in inducing breaks in the cell membrane, leading to an efflux of the internal contents [34]. In a murine model of intestinal mucositis induced by 5-FU, the COG 133 led to a significant reduction in levels of IL-1β and TNF-α and a significant reduction in mRNA levels of iNOS, as well as improvement in intestinal repair and integrity of tight junctions in intestinal cell lines [19]. In another model, ApoE COG133 significantly reduced TNF after 1 h and IL-6 serum levels after 1 and 3 h of LPS challenge [35].

## Conclusion

This study suggest the potential benefit of ApoE COG 133 in ameliorating the effect of TcdA and CDI, which may have clinical implications either as a stand-alone treatment or as an adjunctive intervention with other gut-trophic protective nutrients. Future studies to examine the interplay of these molecules in cell viability mechanism are needed.

## Data Availability

The raw data supporting the conclusions of this article will be made available by the authors, without undue reservation.
